# ^18^F[AlF]-radiolabelled Peptides on the Automated Synthesis Platform: Translating the Laboratory Bench Work to Bedside

**DOI:** 10.21315/mjms2019.26.4.14

**Published:** 2019-08-29

**Authors:** Hishar Hassan, Hairil Rashmizal Abdul Razak, Fathinul Fikri Ahmad Saad, Vijay Kumar

**Affiliations:** 1 Centre for Diagnostic Nuclear Imaging, Universiti Putra Malaysia, Serdang, Selangor, Malaysia; 2 Department of Imaging, Faculty of Medicine and Health Sciences, Universiti Putra Malaysia, Serdang, Selangor, Malaysia; 3 Department of Nuclear Medicine and PET, Westmead Hospital, Westmead, New South Wales, Australia

**Keywords:** ^18^F[AlF] method, radiolabelled peptide, molecular imaging, automated platform

## Abstract

Using radiolabelled peptides that bind, with high affinity and specificity, to receptors on tumour cells is one of the most promising fields in modern molecular imaging and targeted radionuclide therapy (1). In the emergence of molecular imaging and nuclear medicine diagnosis and therapy, albeit theranostic, radiolabelled peptides have become vital tools for in vivo visualisation and monitoring physiological and biochemical processes on molecular and cellular levels (2). This approach may benefit patients in the era of personalised medicine.

## Radiolabelled Peptides in Molecular Imaging

The overexpression of numerous peptide-binding receptors in various tumours and inflammatory tissues have led to the use of radiolabelled peptides for imaging and therapy (2, 3). The cell surface enzyme prostate specific membrane antigen (PSMA), the somatostatin receptor (SST), the bombesin receptor 2 (GRP) and the chemokine receptor (CXCR4) are prominent examples of receptors that are overexpressed as ‘tumour markers’ and linked to tumour development, progression and, often, prognosis, hence inviting researchers to investigate these targets by developing innovative radiopharmaceuticals. Being small, compared to larger targeting compounds like antibodies, makes the peptide an interesting targeting compound because it allows rapid clearance from the blood pool and non-target tissues (2). Additionally, peptides, which are usually non-immunogenic, possess strong tissue penetration properties and high tumour uptake leading to favourable tumour-to-background ratios for excellent image quality and tumour targeting therapy (2, 4).

The radiolabelled peptide debuted in 1989 when Krenning et al. used an ^123^I-radioiodinated somatostatin analogue ([^123^I]204-090) in patients with neuroendocrine tumours (2, 5). Since then, peptides have been labelled with indium-111 (In-111) and technetium-99m (Tc-99m) for single-photon emission computed tomography (SPECT) imaging and with gallium-68 (Ga-68), copper-64 (Cu-64), yttrium-86 (Yt-86) and fluorine-18 (F-18) for positron emission tomography (PET) imaging. For PET imaging modality, F-18 is the most extensively used radioisotope due to its favourable decay properties, highest probability of positron decay, low positron penetration depth and suitable half-life, which allows it to carry out even multistep syntheses and be transported to remote hospitals without an onsite cyclotron (6). On the other hand, the half-life is short enough to avoid extended irradiation for patients (7, 8).

Despite its favourable characteristics, the conventional labelling technique for ^18^F-fluoride often requires harsh reaction conditions, such as high temperatures and the use of polar aprotic organic solvents under basic conditions, which are unsuitable for the labelling of peptides and small proteins (9, 10). Since 2010, continuous research has overcome this limitation by developing attractive alternatives. One such is the introduction of the bifunctional chelator (BFC) suitable for complexation of ^18^F-fluoride bound to a metal and a functional group that allows for bioconjugation to the peptides of interest (1, 6). McBride et al. indicated that fluorine can act as a complexation ligand for Al^3+^, which has been claimed to be stronger than 60 other metal-fluoride bonds (10). The aluminium-fluoride bond (AlF), when in a suitable chemical environment, can be highly stable in vivo and compatible with biological systems (10–15).

Thus, selecting suitable chelators that could stably hold the ^18^F[AlF] complex in such a chemical environment for several hours under physiological, in vivo conditions is a highly attractive alternative to classical F-18 labelling of peptides. Since aluminium (Al^3+^) forms octahedral complexes, the conjugated peptides from triazacyclononane derivatives, such as 1,4,7-triazacyclononane-1,4,7-triacetic acid (NOTA), seem to be ideal candidates for such an approach (1, 10, 16). As a result, direct radiolabelling of peptides with F-18 in a one-step strategy comprising the chelation of the aluminium-fluoride-18 complex (^18^F[AlF]) by the macrocyclic ligand, NOTA, coupled to a peptide, has been investigated and established by several groups (9, 10, 17).

With this available methodology, investigation has already begun to find innovative F-18 labelled peptides, which were previously labelled with the suboptimal radioisotope gallium-68 (Ga-68), such as ^18^F[AlF]-NOTA-octreotides for imaging neuroendocrine tumours, ^18^F[AlF]-NOTA-pentixafor for imaging lymphoproliferative disease and ^18^F-PSMA for imaging prostate cancer (6, 18). Ga-68, with a half-life of only 68 min, is produced from generators that provide limited activity per synthesis, and, depending on the age of the generator, only one to four patient doses per elution can be produced (19). As the radioisotope properties of F-18 are superior to those of Ga-68, and its availability is high, it can be delivered to all PET centres worldwide, together with FDG shipped daily. Therefore, the motivation towards developing ^18^F-labelled peptides is obvious. It allows for producing 18F-labelled peptides via simple processes, in high yields, without necessary investments in expensive Ga-68 generators (20).

## The Dilemma: Translating the Laboratory Bench Work to the Bedside

Unfortunately, despite ^18^F-labelled peptides showing remarkable advantages for receptor imaging and targeted therapy, none of the dedicated, semi-manual or even automated labelling strategies for these peptides, or the NOTA-peptide conjugates themselves, are readily available. Thus, studies on ^18^F[AlF]-NOTA-octreotides, ^18^F[AlF]-NOTA-pentixafor and even ^18^F-PSMA are rare. Most of the required peptide conjugates are only available at dedicated research institutions or centres that can be counted in number and are being backed by strong personnel and research teams. The syntheses are also normally carried out manually by well-trained radiochemists or radiopharmacists.

Thus, a bold move should be considered to close the gap between such high-profile research institutions and ‘normal’ research institutions or university hospitals that provide routine service to patients and are usually operated by nuclear medicine technologists, who prepare the radiopharmaceuticals. This move would include introducing pre-validated synthesis kits and cassettes for radiolabelling peptides on an automated platform or kit base, which could be made accessible to others and made ready to be exploited by other centres with less extensive radiopharmaceutical infrastructures.

## Conclusion

As radiolabelled peptides show remarkable potential in the molecular imaging field, this could promise a new chapter in the era of personalised medicine. Hence, collaborations between stakeholders, researchers and industry players are paramount to making ways for new discoveries and the innovation of pre-validated synthesis kits for radiolabelling peptides on a commercially available, automated platform or kit base, which could be easily handled by technologists.

## Figures and Tables

**Figure 1:**
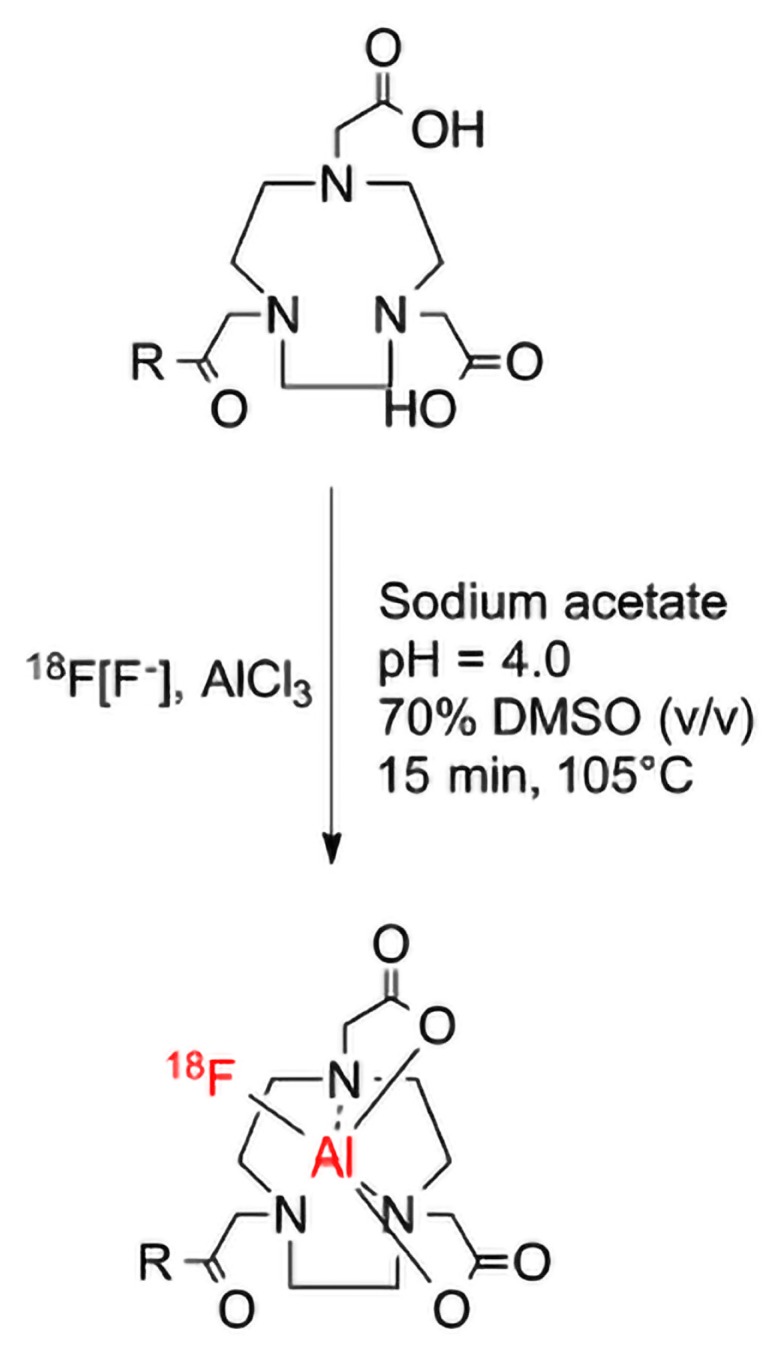
Radiolabelling of pentixafor with F-18 (21)

**Figure 2:**
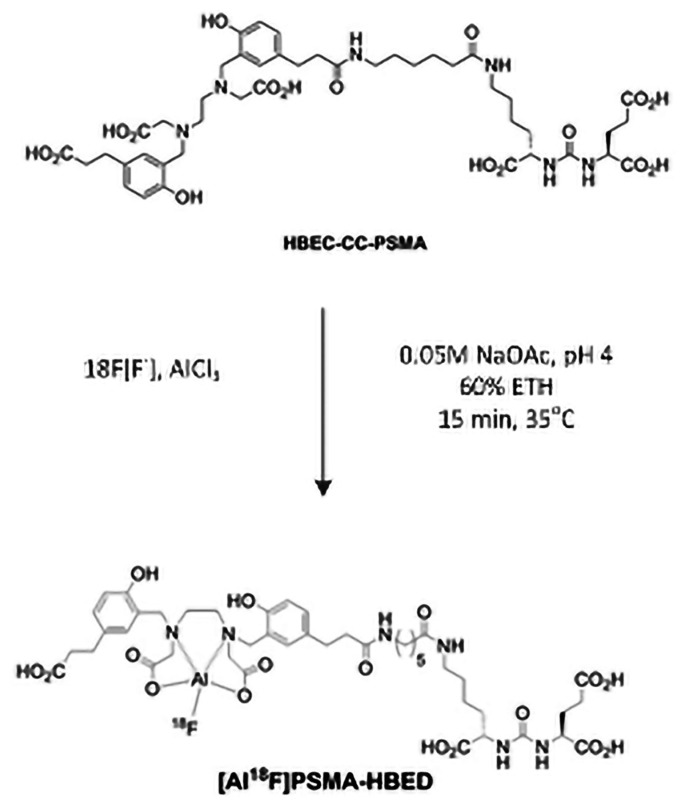
Radiolabelling of PSMA with F-18 (22)
